# How to assess cryoballoon ablation‐related adverse events

**DOI:** 10.1002/joa3.12928

**Published:** 2023-09-19

**Authors:** Naoya Kataoka, Teruhiko Imamura

**Affiliations:** ^1^ Second Department of Internal Medicine University of Toyama Toyama Japan

**Keywords:** atrial fibrillation, arrhythmia, pulmonary vein isolation

## Conflict of interest statement

Authors declare no conflict of interests for this article.

To editor:

Tan and colleagues summarized real‐world clinical data on the adverse events associated with the use of cryoballoon catheters for atrial fibrillation.^1^ They found that phrenic nerve palsy and cardiac perforation were the two most common procedure‐related adverse events. Although the cryoballoon catheter is not a first‐line therapy,^2^ their reports using a large‐scale database are of great importance to many clinicians. Several concerns have been raised to further improve their findings.

The rate of procedure‐related complications is supposed to decline with the innovation of novel devices and improvements in procedural techniques. In another large‐scale study, the procedure‐related complication rate decreased significantly from the early era (2013 and 2017) to the late era (2018 and 2022).^3^ The observation period of the authors' study was between 2011 and 2021,^1^ during which the safety and feasibility of the procedures improved considerably due to the innovation of the second‐generation cryoballoon system. It may be of great interest to analyze the clinical outcomes of the second‐generation devices alone or to compare the devices (i.e., first‐generation versus second‐generation devices).

Phrenic nerve palsy and cardiac tamponade are well‐known procedure‐related complications. Most paralysis is transient. Thus, the novelty of the authors' study is unclear. For example, an intra‐age comparison of the procedure‐related complication rates may be interesting to demonstrate the feasibility of this treatment in elderly patients. A previous study demonstrated a comparable procedure‐related complication rate in elderly patients to the young patients. Observation of natural breathing during the procedure may be a key to early detection of phrenic nerve palsy.^4^ How many patients received cryoballoon treatment under local anesthesia?

## References

[1] Tan MCTJ , Lee WJ , Srivathsam K , Dorajja D , Masry HEI , Scott LR , et al. Adverse events in cryoballoon ablation for pulmonary vein isolation: insight from the Food and Drug Administration manufacturer and user facility device experience. J Arrhythm. 2023. 10.1002/joa3.12898 PMC10549805 37799789

[2] Andrade JG . Ablation as first‐line therapy for atrial fibrillation. Eur Cardiol. 2023;18:e46. 37546183 10.15420/ecr.2023.04 PMC10398511

[3] Benali K , Khairy P , Hammache N , Petzl A , Da Costa A , Verma A , et al. Procedure‐related complications of catheter ablation for atrial fibrillation. J Am Coll Cardiol. 2023;81(21):2089–2099. 37225362 10.1016/j.jacc.2023.03.418

[4] Linhart M , Nielson A , Andrie RP , Mittmann‐Braun EL , Stockigt F , Kreuz J , et al. Fluoroscopy of spontaneous breathing is more sensitive than phrenic nerve stimulation for detection of right phrenic nerve injury during cryoballoon ablation of atrial fibrillation. J Cardiovasc Electrophysiol. 2014;25(8):859–865. 24724724 10.1111/jce.12431

